# Assessing responsiveness of health care services within a health insurance scheme in Nigeria: users’ perspectives

**DOI:** 10.1186/1472-6963-13-502

**Published:** 2013-12-01

**Authors:** Shafiu Mohammed, Justo Lorenzo Bermejo, Aurélia Souares, Rainer Sauerborn, Hengjin Dong

**Affiliations:** 1 Institute of Public Health, Medical Faculty, Heidelberg University, Heidelberg, Germany; 2 Institute of Medical Biometry and Informatics (IMBI), University Hospital Heidelberg, Heidelberg, Germany; 3 Center for Health Policy Studies, Zhejiang University Medical School, Zhejiang, China; 4 Ahmadu Bello University, Zaria, Nigeria

**Keywords:** Health insurance, Users, Health care services, Responsiveness, Nigeria, Performance

## Abstract

**Background:**

Responsiveness of health care services in low and middle income countries has been given little attention. Despite being introduced over a decade ago in many developing countries, national health insurance schemes have yet to be evaluated in terms of responsiveness of health care services. Although this responsiveness has been evaluated in many developed countries, it has rarely been done in developing countries. The concept of responsiveness is multi-dimensional and can be measured across various domains including prompt attention, dignity, communication, autonomy, choice of provider, quality of facilities, confidentiality and access to family support. This study examines the insured users’ perspectives of their health care services’ responsiveness.

**Methods:**

This retrospective, cross-sectional survey took place between October 2010 and March 2011. The study used a modified out-patient questionnaire from a responsiveness survey designed by the World Health Organization (WHO). Seven hundred and ninety six (796) enrolees, insured for more than one year in Kaduna State-Nigeria, were interviewed. Generalized ordered logistic regression was used to identify factors that influenced the users’ perspectives on responsiveness to health services and quantify their effects.

**Results:**

Communication (55.4%), dignity (54.1%), and quality of facilities (52.0%) were rated as “extremely important” responsiveness domains. Users were particularly contented with quality of facilities (42.8%), dignity (42.3%), and choice of provider (40.7%). Enrolees indicated lower contentment on all other domains. Type of facility, gender, referral, duration of enrolment, educational status, income level, and type of marital status were most related with responsiveness domains.

**Conclusions:**

Assessing the responsiveness of health care services within the NHIS is valuable in investigating the scheme’s implementation. The domains of autonomy, communication and prompt attention were identified as priority areas for action to improve this responsiveness. For the Nigerian context, we suggest that health care providers in the NHIS should pay attention to these domains, and the associated characteristics of users, when delivering health care services to their clients. Policy makers, and the insurance regulatory agency, should consider the reform strategies of monitoring and quality assurance which focus on the domains of responsiveness to lessen the gap between users’ expectations and their experiences with health services.

## Background

In recent years, the health systems in most low and middle income countries (LMICs) have changed considerably. Failures in these health systems have led LMICs to devise innovative approaches and alternative mechanisms to improve health service provision. In the last decade, socially oriented national health insurance schemes (NHIS) have been implemented in low LMICs. These health insurance schemes are now widely recognized as alternative means to ensuring an effective and efficient health system for people in LMICs. Several studies have shown that when countries embark on large-scale reforms of their health care systems, periodic monitoring and evaluation are fundamental instruments that ensure the achievement of the initial objectives of reform [1-5]. Responsiveness has been used as a performance outcome indicator in monitoring and evaluating health insurance systems in developed countries, but has received little attention in LMICs.

The responsiveness of health care services to people’s expectations is a target of health insurance schemes which impacts health system goals [2,6]. Responsiveness is included in patient satisfaction and quality of care literature, but is a distinct entity that refers to the way individuals are treated and the environment in which they are treated when seeking health care [7-9]. A responsive health insurance scheme ensures that users are able to obtain healthcare in a client-oriented manner with no discrimination of different population groups [2,6,9]. This is increasingly important considering the set of responsiveness domains proposed by World Health Organization (WHO) in 2000. These domains, including patients’ experiences with prompt attention, dignity, communication, autonomy, choice of provider, quality of facilities, confidentiality and access to family support [9], are essential in evaluating the performance of insurance schemes and the impacts on health systems’ goals, at all stages of development [2]. Although most studies exploring responsiveness related to patient experience and expectations are from high income countries, especially Western Europe, there is growing interest in evaluating the people’s experience with health care services in LMICs [2,8,10].

Evaluating how the insurance system responds to users’ expectations, related to health care services during the implementation process, enables policy and decision makers to better understand the scheme, so that midcourse corrections can be made in a timely and effective manner [1-3,5]. In addition, evidence derived from assessing the system’s responsiveness contributes to providers’ accountability, which can augment end-users’ support and confidence in the reform initiatives [1-3,5]. This evidence helps guide allocation of resources, by showing which domains are most important and critical to improve, at the same time as allowing policy development specific to national, regional, or population group needs [5,10]. However, the function of these health insurance schemes, in terms of responsiveness, has been given little attention in LMICs. One of the purposes of health financing schemes, as stated by WHO, is to ensure that individuals have access to effective public health and personal health care [2,6,9]. One of the advantages of health insurance is securing and improving health care services [11] so that the NHIS can meet some of its objectives [12-14] regarding access to- and delivery of good health care services.

As part of the health sector reform programme in Nigeria, the national health insurance scheme (NHIS) was established by federal law in 1999. The NHIS aims to provide health insurance, which entitles insured-users and their dependants to good quality and cost-effective healthcare services [15]. With an expansion strategy to achieve universal coverage, the NHIS has been divided into four broad programmes including: formal sector, informal sector, vulnerable groups and others (international travel health insurance, retirees, and unemployed) [15]. In 2005, the NHIS was introduced into all federal establishments in Nigeria through the formal sector social health insurance programme (FSSHIP). Presently, the health insurance is based on a single fund, similar to that of Tanzania, and has concluded its first phase of implementation, including only federal civil servants. The scheme has already shifted to the second phase of implementation, extending to include states’ civil servants and the informal sector. The FSSHIP covers formal sector employees, including federal, state, and local governments, and organized private institutions with at least 10 employees [12-14]. Nevertheless, the Nigerian government’s strategy is to bring all four programmes on board through a phased approach. The NHIS has only recently started the informal-sector programme, with pending unresolved issues. This scheme is intended to provide financial protection, improved health care delivery and utilization of equitable and affordable health services through contributions, risk pooling and purchasing of services.

Within the NHIS in Nigeria, services are purchased from a mix of public and private providers and reimbursed by purchasing-agency resources controlled by health management organizations (Hmos). These Hmos are insurers who, vertically integrated in the scheme’s revenue collection, pool and purchase healthcare services within a competitive framework. This hybrid model of operation creates a purchaser-provider split and the health insurers operate for-profit or not-for-profit in the insurance scheme. The Hmos are private organizations which coordinate all aspects of the delivery system and manage reimbursement to accredited HCPs [16]. Furthermore, the Hmos have contracts with independently existing HCPs to provide health care services to their insured clients. In principle, a client can chose any HCP, and the Hmo initiates a contract with the client’s provider. In practice, these HCPs independently exist as either public or private health facilities, including hospitals and clinics. Presently, there are 62 Hmos in Nigeria licensed by the NHIS regulatory agency, which facilitates the interface between governmental organizations, health care providers, and eligible contributors (JLN 2011).

Although the Nigerian NHIS aims to create access to good quality and cost-effective healthcare services [12-15], it has been reported that insured-persons have complained of service providers with poor attitudes [12,17]. Problems with responsiveness in health care service provision need to be understood and rapidly resolved. Monitoring and evaluating health service provision and health care providers’ activities would help identify areas for improvement during future implementation strategies.

This study focused on the responsiveness of health care services to users’ expectations as an outcome indicator of the performance of the NHIS in Nigeria. In this study, responsiveness refers to the measurement of how the health insurance scheme performs the non-therapeutic aspects of health related activities that not only affect the users’ experience of health care service provision but also impact health outcomes [9,10,18,19]. To our knowledge, this is the first in-depth study that examines responsiveness domains and methodological view-point in the context of evaluating the performance of a health insurance schemes in an LMIC. It is essential to consider how the NHIS has impacted the barriers to health care utilization, perceived quality of care and access to information [20]. Policy and decision makers need to understand the factors influencing the users’ perspectives on the responsiveness domains in order to effectively implement such health insurance schemes [19]. However, responsiveness may also depend on factors beyond the health insurance system. Here, we focussed on responsiveness of health care services to users’ expectations and experience, and individual level determinants. This study was carried out to evaluate the progress of implementation of the NHIS related to responsiveness of health care services.

We hypothesized that users of the health insurance scheme have differences in their expectations of the various responsiveness domains and their ranking of importance, based on certain individual factors and encounters with health care providers (HCPs). We assumed that certain user groups would experience less responsiveness, including: users of public health facilities, the elderly, females, users with lower educational status, low salary income earners, users with longer duration of enrolment, users with fewer visits to providers, and users with referral for secondary or tertiary care after enrolment. Moreover, we also explored polygamous status as a cultural-traditional values proxy and its influence on the perspectives of the responsiveness domains. Our hypothesis, in respect to these assumptions, was supported by the theoretical framework of various responsiveness reviews, debates and adaptations [2,9,10,21,22]. Our study substantiates the importance of responsiveness domains and supported the theoretical framework of performance indicators for the implementation of social health insurance schemes [2,6].

## Methods

### Study setting

This retrospective cross-sectional survey was conducted in Kaduna State between October 2010 and March 2011. Kaduna State is located in the central region of Nigeria, bordering the federal capital territory (Abuja). The distance between Kaduna (state capital) and Abuja is 186 kilometres. There are 23 local government areas in the state. Primary and secondary health facilities are located in each local government area. The state has 4 tertiary health care facilities. At the time of the study, Kaduna State had one of the largest numbers of enrolees in the NHIS (159,789) consisting of principals (48,944) and dependants (110,845) as identified by the NHIS regulatory agency. In Nigeria, the NHIS specified that “contributions made by an insured-person (principal enrolee) entitle him, his spouse and four biological children under the age of 18 years to a defined health benefit-package” [13,15]. Most of the health facilities in the state rendered health services to enrolees, and have been accredited since the inception of the national health insurance scheme in Nigeria.

### Sampling

Two-staged sampling was used to select the study participants. In the first stage, 39,541 principal enrolees, who were insured for more than one year in the insurance scheme, were selected from among the 68 health facilities that had been accredited by the NHIS during the three years preceding the survey (NHIS 2010 regional office data, Kaduna). The second stage involved random selection of those principal enrolees who visited the health facilities during the time of the survey. Participants were selected using a random systematic sampling scheme with a randomized start from patients’ daily lists that were compiled as part of the study. Non-responsive enrolees were replaced during the survey to ensure facility-size representation. Verification of health facilities and enrolees was carried out in collaboration with the NHIS regional officers in Kaduna and NHIS headquarters in Abuja-Nigeria. Only enrolees who met the inclusion criteria at both stages were included in the study, resulting in 796 insured users (IUs) from 57 accredited health facilities, who were then interviewed face-to-face. The inclusion criteria were that the participant must (i) have been insured at least for 1 year in the insurance scheme, and (ii) have used a health facility accredited by the NHIS during the three years preceding the survey. This was done to ensure that the participants had adequate knowledge, experience and interactions with the NHIS and their accredited providers in the insurance system [17].

### Questionnaire

A pre-tested, interviewer-administered questionnaire was used. The questionnaire covered the users’ characteristics related to their perceptions of the responsiveness domains. Questionnaire manuals, which defined various key terms and responsiveness domains, were incorporated as addenda for the participants, in order to enhance clarity and to limit misinterpretation of terms by the respondents. The study used a modified section of a short, out-patient responsiveness questionnaire included in the 2002–2003 World Health Survey [23], designed by the World Health Organization (WHO) for measuring health system performance [the survey instrument is available at: http://www.who.int/responsiveness/surveys/individual.pdf].

The questionnaire was modified to collect basic information about respondents’ (insured users) characteristics related to the health insurance scheme. The explanatory variables were selected based on the study objectives and integrated hypotheses and included respondent’s age, sex, education, income, type of facility visited, marital status, duration of enrolment, referral, and contact with providers. These variables were derived from a review of related extant literature [9,10,17,21,22,24-26]. For example, we were interested in investigating the effect of polygamy as a socio-cultural norm, previous contact with providers, and users’ experience with referrals in the insurance scheme. In this study, we included users’ referral after enrolment in the explanatory variables, which could provide additional information on their experiences with the insurance scheme.

Responsiveness domains were measured using a Likert-type scale of five ordered categories which were rated from one (very good) to five (very bad). However, to improve the statistical plausibility and provide a better fit with the models used, the ordered categories were reversed from one (very bad) to five (very good) during the analysis. To determine responsiveness, respondents were asked to rate their experience of six domains during their most recent contact with the health care services provided in the insurance scheme. These included “prompt attention” (short waiting times), “dignity” (respectful treatment), “communication” (clarity of communication by providers), “autonomy” (involvement in decisions), “choice of provider” (patient choice of providers), and “quality of basic facilities” (clean facilities for patients’ convenience). The definitions of these responsiveness domains were adapted from the WHO model of evaluating responsiveness based on users of the health insurance system [9,10] and are presented in Table 1. We excluded the domains of “access to family support” and “confidentiality” because our study focused on users’ outpatient experiences. Furthermore, our piloted study showed that the users’ understanding of “confidentiality” was limited by the providers’ rules preventing users having access to their personal health records and discouraging users’ questions or comments regarding other health staff’s intrusions during users’ physical examinations.

**Table 1:** Definitions of responsiveness domains

**Domain name**	**Definition (also provided in the interview manual)**
Prompt attention	This meant patients have short travel times and convenient access to health care facilities. They obtain fast care in emergencies and have short waiting times for appointments and consultations. Tests get done quickly.
Dignity	This is termed patients are shown respect by greetings before talking to them. Physical examinations are conducted in privacy and in a way that respects their cultural norms.
Communication	This meant the provider listens carefully to patients and explains things so patients can understand. Patients have time to ask questions, if they don’t understand something.
Autonomy	This is termed patients are involved in deciding on their treatment if they want to. The provider asks patients for permission before starting treatments or tests.
Choice	This meant patients are able to choose their health care provider (place or person) and allowed to consult for a second opinion or with a specialist, if so desired.
Quality of facilities	This refers to having enough space, seating places and fresh air in the waiting and examination rooms as well as hospital wards. It includes a clean facility and clean toilets in the hospital.

Adapted from Valentine et al. 2002 and WHO 2000.

### Statistical analysis

We analysed the responsiveness of the NHIS health care services using six responsiveness domains. We assessed each domain and its importance as a Likert-type ordered category. However, the ordered category was reversed from one (very bad) to five (very good) during the analysis. Generalized ordered logistic regression was used to model the influential factors associated with perspectives on the responsiveness domains. After testing for collinearities, no variable-pair showed a correlation above 0.75 that required elimination.

The statistical model used to analyze the health care services responsiveness domains examines ordinal outcomes, which conform or violate the proportional odds assumptions. The regression used is a generalization of the logit analysis for the case of more than two outcomes of an ordinal-dependent variable. The latent evaluation score *y_*i*_ is a linear function of independent variables vector *x*_*i*_ where the *i* depicts individual observation,

$$*y_{i}=x_{i}\prime \beta +\epsilon_{i}$$

Where *β* is a coefficient vector, and *ϵ*_*i*_ is the random error for the i-th individual assumed to follow a standard normal distribution. The data for the i-th individual in the design matrix is represented by *x*_*i*_ and we assume the response *y_*i*_ (for the responsiveness domains) is unobserved. Researchers observe *y*_*i*_ for each of the domains through the following:

$$\mathrm{yi}=\left\{\begin{array}{l} 1\;\mathrm{if}‒\infty \leq *\mathrm{yi}<{µ}_{1}\\ 2\;\mathrm{if}\;{µ}_{1}\leq *\mathrm{yi}<{µ}_{2}\\ 3\;\mathrm{if}\;{µ}_{2}\leq *\mathrm{yi}<{µ}_{3}\\ 4\;\mathrm{if}\;{µ}_{3}\leq *\mathrm{yi}<{µ}_{4}\\ 5\;\mathrm{if}\;{µ}_{4}\leq *\mathrm{yi}<\infty \end{array}\right)$$

The cut points (i.e., threshold values) μ_1,_ μ_2,_ μ_3,_ μ_4_ are unknown parameters for each domain to be estimated based on the maximum likelihood for the ordered logit. The parallel-lines model fitted by the ordered logit model is expressed as:

$$\begin{array}{l} P\left(Y_{i}>j\right)=g\left(\mathrm{X\beta}\right)=\frac{\exp\left(\alpha_{j}+X_{i}\beta \right)}{1+\left[\exp\left(\alpha_{j}+X_{i}\beta \right)\right]},\\ \;\;j=1,2,3,4 \end{array}$$

where, the *β*_’s_ (but not the *α*_’s_) are the same for all values of j. Here, j refers to the level of the response variable, with j = 1 as the reference category. Since it is common for one or more *β*_’s_ to differ across values of j, we applied a more flexible model that fits three special cases of the generalized model: the *proportional odds model,* the *partial proportional odds model,* and the *logistic regression* model [27-29]. This model is presented by:

$$\begin{array}{l} P\left(Y_{i}>j\right)=g\left(\mathrm{X\beta}\right)=\frac{\exp\left(\alpha_{j}+X_{i}\beta_{j}\right)}{1+\left[\exp\left(\alpha_{j}+X_{i}\beta_{j}\right)\right]},\\ \;\;j=1,2,3,4 \end{array}$$

The probabilities that Y will take on each of the values 1, 2, 3, 4, 5 are equal to:

$$\begin{array}{l} P\left(Y_{i}=1\right)=1-g\left(\mathrm{Xi}\beta_{1}\right)\\ P\left(\mathrm{Yi}=j\right)=g(\mathrm{Xi\beta j}-1)-g(\mathrm{Xi\beta j})\;j=2,3,4\\ P\left(Y_{i}=5\right)=g(\mathrm{Xi}\beta_{4}) \end{array}$$

An advantage of this generalized ordered logistic regression is its flexibility regarding parallel-lines assumptions, and its avoidance of inflation of estimates. Associations between the outcome and independent variables were assessed using coefficient and probability values. “Impact” is defined as the magnitude of each independent variable on the dependent variable. It implies the absolute change in odds (*e*^|coef.|^) over the entire range of the independent variable. A p-value of 0.05 was used as the threshold for statistical significance. In the multiple-generalized ordered logistic regression model, the response variables included prompt attention, dignity, communication, autonomy, choice of provider and quality of facilities. The list of independent variables is shown in Table 2. Computations were performed using the gologit2 function of STATA®. STATA program (STATA® 12.1, 2011; StataCorp LP., 4905 Lakeway Drive, College Station, Texas, 77845 USA) was used to carry out all analyses.

**Table 2:** Characteristics of insured users (respondents) to investigate health service responsiveness

**Independent variables**	**Categories**	**Frequency**	**Percentage**
Type of facility visited	Public	435	54.7
	Private	361	45.3
Age	<40 years	387	48.6
	≥40 years	409	51.4
Sex	Male	431	54.2
	Female	365	45.8
Educational status^1^	Lower education	247	31.0
	Higher education	549	69.0
Monthly income level (US$)^2^	≤187 dollars	239	30.0
	>187 dollars	557	70.0
Type of marital status	Polygamy	187	23.5
	Others^3^	609	76.5
Duration of enrolment	≤2 years	282	35.4
	>2 years	514	64.6
12 months visits to HCPs^*^	1–3 visits	231	29.0
	>3 visits	565	71.0
Referral after enrolment^4^	Yes	363	45.6
	No	433	54.4
Total N (%)		796	100

^*^HCPs = health care providers.

^1^Lower education = secondary school and below; higher education = polytechnics and universities.

^2^Up to 187 US dollars is ~30000 naira (Nigerian currency) considered as lowest government-approved monthly salary, all as at the time of the study.

^3^Includes monogamy, those not yet married, divorced and widowed because we are interested in investigating the effect of polygamy as a socio-cultural norm.

^4^Insured-users that had ever been referred for secondary or tertiary health care in the insurance scheme.

The study protocol was approved by the Ethics Commission of Heidelberg University-Germany [S-035/2009], University Research Ethics Committee ABU-Nigeria [VC/P. 18890] and the NHIS Headquarters Abuja-Nigeria [NHIS-467].

## Results

### Health insurance users’ (respondents) characteristics, expectations and experience related to responsiveness of health care services

The insured users’ (respondents) characteristics, related to their perceptions of the NHIS responsiveness to health care services, are presented in Table 2. Among the 1000 insured users (IUs) approached, 796 responded (i.e. response rate was 79.6%). More than half of the respondents were users of public health facilities (54.7%). The majority were males, with a higher education and monthly salary income above 187 US dollars (30,000 naira). Note, the thirty-thousand naira monthly salary is considered among the lowest earnings within the government approved salary scale levels. A majority of the respondents (64.6%) had been enrolled for at least 2 years in the NHIS and had at least three visits to their health facility in the last twelve months preceding the survey. However, less than half of the respondents (45.6%) had ever been referred for secondary or tertiary care after their enrolment in the NHIS, with a further minority of 23.5% that had a polygamous family.

The importance that respondents attached to various responsiveness domains of health service provision is presented in Table 3. The interviewees rated the importance of each domain using an ordered Likert scale, with 5 categories from “extremely important” to “not important”. We describe only the modal responses to facilitate easy interpretation. If we concentrate on the predominant responses for each domain, “prompt attention”, “autonomy” and “choice of provider” were considered “very important”, but “dignity”, “communication” and “quality of facilities” were perceived as “extremely important”.

**Table 3:** Importance attributed by insured users (respondents) to responsiveness domains

**Domains responses**	**Prompt attention travel and short waiting times (%)**	**Dignity respectful treatment (%)**	**Communication clarity of communication (%)**	**Autonomy involvement in decision making (%)**	**Choice of provider patient choice of providers’ (%)**	**Quality of facilities good quality of surroundings (%)**
Extremely important	307 (38.6)	431 (54.1)	441 (55.4)	278 (34.9)	319 (40.1)	414 (52.0)
Very important	365 (45.9)	312 (39.2)	319 (40.1)	366 (46.0)	372 (46.7)	296 (37.2)
Moderately important	99 (12.4)	35 (4.4)	19 (2.4)	111 (13.9)	83 (10.4)	66 (8.3)
Slightly important	16 (2.0)	10 (1.3)	9 (1.1)	29 (3.7)	11 (1.4)	11 (1.4)
Not important	9 (1.1)	8 (1.0)	8 (1.0)	12 (1.5)	11 (1.4)	9 (1.1)
Total	796 (100)	796 (100)	796 (100)	796 (100)	796 (100)	796 (100)

The IUs (respondents) responses, based on their experiences, and the responsiveness domains assessed are presented in Table 4. The domains were assessed and reported according to 5 categories on a Likert-scale ranging from “very good” to “very bad”. If only the modal response of each domain is considered, all of them were rated as “good”. However, there were variations in their respective rating percentages with “quality of facilities” ranking highest whereas “communication” and “autonomy” were relatively the lowest.

**Table 4:** Experiences (ratings) of insured users (respondents) of responsiveness domains

**Domains responses**	**Prompt attention travel and short waiting times (%)**	**Dignity respectful treatment (%)**	**Communication clarity of communication (%)**	**Autonomy involvement in decision making (%)**	**Choice of provider patient choice of providers’ (%)**	**Quality of facilities good quality of surroundings (%)**
Very good	230 (28.9)	308 (38.7)	254 (31.9)	172 (21.6)	222 (27.9)	320 (40.2)
Good	314 (39.5)	337 (42.3)	304 (38.2)	299 (37.6)	324 (40.7)	341 (42.8)
Moderate	199 (25.0)	96 (12.1)	165 (20.7)	221 (27.7)	189 (23.8)	92 (11.6)
Bad	36 (4.5)	31 (3.9)	53 (6.7)	71 (8.9)	32 (4.0)	19 (2.4)
Very bad	17 (2.1)	24 (3.0)	20 (2.5)	33 (4.2)	29 (3.6)	24 (3.0)
Total	796 (100)	796 (100)	796 (100)	796 (100)	796 (100)	796 (100)

### Health insurance users’ factors that influences responsiveness domains

The results obtained in this section are analysed in Tables 5 and 6 according to the generalized ordered logistic regression model [Additional file 1 as DOCX]. Calculations are depicted for IUs’ characteristics in relation to their reported experiences (or ratings) of the responsiveness domains.

**Table 5:** **User’s characteristics and their relation with experiences of responsiveness domains**^**1**^

**Independent variables**	**Prompt attention**^**1**^				**Dignity**^**1**^				**Communication**^**1**^			
	**Coef.**	**SE**	**p-value**	**Impact**	**Coef.**	**SE**	**p-value**	**Impact**	**Coef.**	**SE**	**p-value**	**Impact**
Type of facility visited												
Private (ref.)												
Public	−0.68	0.14	<0.001	0.51	−0.67	0.14	<0.001	0.51	−1.31^a^	0.63	0.039	0.27
									−0.02^b^	0.25	0.923	1.02
									0.56^c^	0.17	<0.001	1.55
									0.60^d^	0.16	<0.001	1.57
Age												
≥40 years (ref.)												
<40 years	−0.06	0.14	0.693	0.94	−0.01	0.15	0.968	0.99	0.02	0.15	0.895	1.02
Sex												
Female (ref.)												
Male	0.38	0.13	0.004	1.47	0.27	0.13	0.042	1.32	0.21	0.13	0.123	1.23
Educational Status												
Higher education (ref.)												
Lower education	0.20	0.15	0.217	1.22	−0.33	0.16	0.033	0.71	−0.05	0.16	0.759	0.95
Monthly income level												
>187 US dollars (ref.)												
≤187 US dollars	0.24	0.16	0.140	1.27	0.06	0.16	0.691	1.06	0.36	0.16	0.029	1.43
Type of marital status												
Others (ref.)												
Polygamy	−0.07	0.15	0.678	0.94	0.04	0.17	0.818	1.04	−0.30	0.16	0.063	0.74
Enrolment duration												
>2 years (ref.)												
≤2 years	0.54^a^	0.59	0.365	1.71	0.18	0.14	0.216	1.19	0.24^a^	0.55	0.665	0.99
	0.06^b^	0.30	0.837	1.06					0.31^b^	0.26	0.964	1.27
	0.44^c^	0.17	0.012	1.55					0.35^c^	0.18	<0.001	1.42
	−0.07^d^	0.17	0.684	0.93					0.63^d^	0.16	0.033	1.89
12 months visits to HCPs^*^												
>3 visits (ref.)												
1–3 visits	−0.55^a^	0.55	0.320	0.58	0.23	0.14	0.110	1.26	−0.32^a^	0.46	0.484	0.73
	−1.12^b^	0.37	0.003	0.33					−0.05^b^	0.26	0.849	0.95
	−0.45^c^	0.17	0.008	0.63					−0.66^c^	0.18	<0.001	0.51
	−0.16^d^	0.17	0.360	0.85					0.12^d^	0.17	0.489	1.13
Referral after enrolment												
No (ref.)												
Yes	−0.05^a^	0.49	0.918	0.95	0.88^a^	0.46	0.053	2.42	−1.28^a^	0.52	0.013	0.28
	0.28^b^	0.29	0.329	1.33	0.39^b^	0.29	0.170	1.48	−0.31^b^	0.25	0.220	0.74
	0.49^c^	0.16	0.002	1.57	−0.06^c^	0.18	0.729	0.94	−0.73^c^	0.16	<0.001	0.48
	0.55^d^	0.17	0.001	1.61	−0.42^d^	0.15	0.006	0.66	−0.84^d^	0.16	<0.001	0.43
Constant	4.17^a^	0.59	<0.001	64.72	3.27^a^	0.31	<0.001	26.31	5.28^a^	0.78	<0.001	196.18
	3.39^b^	0.40	<0.001	29.62	2.56^b^	0.26	<0.001	12.95	2.22^b^	0.32	<0.001	9.24
	1.18^c^	0.23	<0.001	3.27	1.60^c^	0.22	<0.001	4.94	1.54^c^	0.24	<0.001	4.67
	−0.58^d^	0.21	0.007	0.55	−0.24^d^	0.21	0.024	0.78	−0.60^d^	0.22	0.005	0.54

^*^HCPs = health care providers.

^1^Dependent variable coding: (1) Very bad; (2) Bad; (3) Moderate; (4) Good; (5) Very good. For variables that violate the proportional odds assumption.

^a^Coefficient for response category (1) contrasted with categories (2), (3), (4), and (5).

^b^Coefficient for response categories (1) and (2) contrasted with categories (3), (4), and (5).

^c^Coefficient for response categories (1), (2), and (3) contrasted with categories (4) and (5).

^d^Coefficient for response categories (1), (2), (3), and (4) contrasted with category (5).

Impact is calculated as the absolute change in odds (*e*^|coef.|^).

**Table 6:** **User’s characteristics and their relation with experiences of responsiveness domains**^**1**^

**Independent variables**	**Autonomy**^**1**^				**Choice of provider**^**1**^				**Quality of facilities**^**1**^			
	**Coef.**	**SE**	**p-value**	**Impact**	**Coef.**	**SE**	**p-value**	**Impact**	**Coef.**	**SE**	**p-value**	**Impact**
Type of facility visited												
Private (ref.)												
Public	1.16^a^	0.43	0.006	3.20	−0.36	0.13	0.008	0.70	−0.92	0.14	<0.001	0.40
	0.85^b^	0.22	<0.001	2.34								
	−0.34^c^	0.15	0.025	0.71								
	−0.41^d^	0.17	0.020	0.66								
Age												
≥40 years (ref.)												
<40 years	0.69^a^	0.39	0.074	1.99	−0.23	0.14	0.107	0.79	0.04	0.15	0.765	1.05
	0.41^b^	0.22	0.067	1.51								
	−0.15^c^	0.16	0.367	0.86								
	−0.17^d^	0.18	0.361	1.19								
Sex												
Female (ref.)												
Male	0.25	0.13	0.057	1.28	−0.14^a^	0.37	0.708	0.87	0.09	0.14	0.522	1.09
					−0.08^b^	0.27	0.764	0.92				
					0.02^c^	0.16	<0.001	2.04				
					0.17^d^	0.16	0.883	1.02				
Educational Status												
Higher education (ref.)												
Lower education	0.33^a^	0.44	0.453	1.39	0.14	0.15	0.363	1.16	−0.37	0.16	0.025	0.69
	−0.21^b^	0.24	0.378	0.81								
	−0.36^c^	0.17	0.041	0.44								
	−0.06^d^	0.20	0.754	0.93								
Monthly income level												
>187 US dollars (ref.)												
≤187 US dollars	0.20	0.16	0.220	1.21	0.29	0.16	0.073	1.34	0.69	0.17	<0.001	1.70
Type of marital status												
Others (ref.)												
Polygamy	−0.39	0.16	0.014	0.68	−0.32	0.16	0.044	0.72	−0.11	0.16	0.505	0.90
Duration of enrolment												
>2 years (ref.)												
≤2 years	0.33	0.14	0.019	1.39	0.49	0.14	0.001	1.62	0.03	0.15	0.836	1.03
12 months visits to HCPs^*^												
>3 visits (ref.)												
1–3 visits	−0.14	0.14	0.315	0.87	0.61^a^	0.37	0.106	1.83	0.23^a^	0.42	0.578	1.26
					1.06^b^	0.27	<0.001	2.90	0.37^b^	0.32	0.241	1.45
					0.16^c^	0.17	0.343	1.17	−0.47^c^	0.21	0.028	0.63
					0.32^d^	0.18	0.074	1.38	−0.25^d^	0.17	0.128	0.77
Referral after enrolment												
No (ref.)												
Yes	0.28^a^	0.41	0.501	1.32	1.03^a^	0.44	0.019	2.81	−0.81	0.14	<0.001	0.44
	0.18^b^	0.22	0.408	1.20	0.47^b^	0.27	0.081	1.60				
	−0.34^c^	0.15	0.023	0.71	−0.39^c^	0.16	0.014	0.67				
	−0.57^d^	0.18	0.002	0.56	−0.65^d^	0.17	<0.001	0.52				
Constant	1.98^a^	0.35	<0.001	7.26	2.71^a^	0.41	<0.001	15.09	4.10^a^	0.36	<0.001	60.34
	1.12^b^	0.24	<0.001	3.09	1.84^b^	0.29	<0.001	6.30	3.40^b^	0.29	<0.001	30.11
	0.50^c^	0.21	0.015	1.66	0.51^c^	0.22	0.020	1.66	2.58^c^	0.25	<0.001	13.17
	−1.12^d^	0.23	<0.001	0.32	−0.96^d^	0.23	<0.001	0.38	0.22^d^	0.21	0.029	1.25

^*^HCPs = health care providers.

^1^Dependent variable coding: (1) Very bad; (2) Bad; (3) Moderate; (4) Good; (5) Very good. For variables that violate the proportional odds assumption.

^a^Coefficient for response category (1) contrasted with categories (2), (3), (4), and (5).

^b^Coefficient for response categories (1) and (2) contrasted with categories (3), (4), and (5).

^c^Coefficient for response categories (1), (2), and (3) contrasted with categories (4) and (5).

^d^Coefficient for response categories (1), (2), (3), and (4) contrasted with category (5).

Impact is calculated as the absolute change in odds (*e*^|coef.|^).

In the prompt attention domain, depicted in Table 5, IUs who received care from the public providers were less likely to express that they received prompt attention than those receiving care from the private providers (p < 0.001; impact = 0.51). Males reported higher prompt attention compared to females (p = 0.004; impact = 1.47). The IUs that were ever referred for secondary or tertiary care were much more likely to report high levels of prompt attention than those who never had a referral in the NHIS. IUs’ type of health facility visited, age, sex, education, monthly income level and type of marital status conformed to proportional odds assumptions.

As presented in Table 5 related to the dignity domain, IUs who received care from public providers were less likely to report being treated with dignity than those receiving care from private providers (p < 0.001; impact = 0.51). Males were more likely to report higher dignity scores than females (p = 0.042; impact = 1.32). IUs with a relatively low educational status reported lower dignity scores compared to those with better education (p = 0.033; impact = 0.71). Here, only the IUs referral after enrolment violated the proportional odds assumption.

The relation of IUs’ characteristics to the communication domain is also depicted in Table 5. IUs who consulted public providers were much more likely to report high levels of better communication than those who consulted private providers in the NHIS. Poor people were more likely to report better communication than the rich people (p = 0.029; impact = 1.43). We observed a negative effect on IUs with a polygamous family for the communication domain, but only at significance level less than 0.1 (p = 0.063; impact = 0.74). IUs with up to two years of enrolment in the NHIS were much more likely to report high levels of better communication and this effect consistently increased across the range of communication. On the other hand, IUs who were ever referred for secondary or tertiary care were much less likely to report high levels of better communication. Here, type of facility visited by IUs, their duration of enrolment in the NHIS, 12 month visits to providers, and referral after enrolment in the scheme violated the proportional odds assumption.

In Table 6, IUs who received care from public providers were much more likely to report low levels of autonomy and this effect consistently decreased across the range of autonomy. Males were more likely to experience better autonomy than females, although only with a significance at less than 0.1 (p = 0.057; impact = 1.28). By contrast, IUs with a polygamous family were less likely to report better autonomy than those living in monogamy or being not yet married (p = 0.014; impact = 0.68). IUs with up to two years of enrolment in the NHIS reported better autonomy as compared to those with at least two years of enrolment (p = 0.019; impact = 1.39). The IUs who were ever referred for secondary or tertiary care were much more likely to report high levels of autonomy and this effect consistently decreased across the range of autonomy.

The choice of provider domain is analysed in Table 6. The IUs who received care from public providers were less likely to report a good choice of provider than those receiving care from private providers (p = 0.008; impact =0.70). Low income IUs reported better choice of provider than high income IUs, but only with a significance level less than 0.1 (p = 0.073; impact = 1.34). By contrast, IUs with a polygamous family were less likely to report better choice of providers than those living in monogamy or being not yet married (p = 0.044; impact = 0.72). The IUs with at most two years of enrolment in the NHIS reported better choice of provider than those with at least two years of enrolment (p = 0.001; impact = 1.62). The IUs who were ever referred for secondary or tertiary care were much more likely to report low levels of autonomy and this effect consistently decreased across the range of autonomy.

The quality of facilities as perceived by the IUs is analysed in Table 6. IUs who received care from public providers were less likely to perceive better quality of facilities than those receiving care from private providers (p < 0.001; impact = 0.40). Furthermore, IUs with relatively low education were less likely to report better quality of facilities than those with higher education (p = 0.025; impact = 0.69). By contrast, low income IUs reported better quality of facilities than high income IUs (p < 0.001; impact = 1.70). The IUs that were ever referred for secondary or tertiary care were less likely to have perceived better quality of facilities than those who never had referral in the NHIS (p < 0.001; impact = 0.44). Here, by contrast, only IUs with visits to providers during the 12 previous months violated the proportional odds assumption.

## Discussion

The IUs’ experiences of the responsiveness of health care services within the NHIS were assessed in this study, according to several responsiveness domains. The performance of each domain during the implementation period (since 2005) of the NHIS was determined and the relative importance of each was also investigated. Scientifically, there is a tradition of both evaluating responsiveness and assessing the importance of its related domains when studying the health care services of any health system or program [9,10]. The importance attached to each domain by the users might augment their respective expectations, which are, in turn, contextually related to their country’s general perception [9,10]. Furthermore, the IUs’ factors and concerns, potentially related to responsiveness domains, were examined based on their experiences with the NHIS. Principal enrolees with more than one year in the insurance scheme were considered to have relevant experiences that could provide information on the responsiveness domains. The findings of this study are discussed according to the responsiveness domains.

### Prompt attention

The domain of prompt attention was poorly rated by the IUs. Valentine et al. [10] explained that this domain covers people’s experience with access to rapid care and short waiting periods for treatment. Previous studies have shown that lack of prompt attention by providers, due to delays in administrative processes and settling of insurance claims, negatively affects the IU’s encounter with health care services [9,10,24]. The evidence from our findings suggests that the NHIS should ensure that IUs receive the necessary healthcare within an appropriate time period, either in public or private health facilities. Active monitoring might help promote and enhance prompt attention to the IUs.

Our findings are similar to those of other studies in Nigeria, Ghana and South-Africa where the type of facility was found to influence prompt attention: the public providers performed poorly in the domain of prompt attention compared to private providers [25,26,29]. Several studies explain that the poor performance of public providers is attributable to giving patients an appointment for a particular day without a specific patient consultation time [25], high patient numbers exceeding the capacity of public facilities [25], poor quality of public services [30], especially poor attitude of providers versus the insured-users and bad interpersonal relationships [17,29].

Male IUs were found in our study to rate the prompt attention domain higher than female IUs. In a male dominated society like Nigeria, a possible explanation may be that males are given priority over females during demand for services. If this interpretation is correct, attention should be focused on females receiving equal priority in these services. We found the likelihood that, IUs who had ever been referred for secondary or tertiary care rated higher in the prompt attention domain. This may not be surprising, because a cross-countries analysis by Valentine et al. [10] suggested the possibility that people who were referred for secondary or tertiary care might have been favoured by the providers.

### Dignity

In Nigeria, dignity of users is explicitly considered as an important element of responsiveness within health care services [25]. Similarly, we found users of health care services rated their contentment with this domain second highest in the NHIS. Similar to the World Health Report 2000, Valentine et al. [10] revealed that the domain of dignity assures users of health care services receive care in a respectful, caring and non-discriminative manner. Generally, our findings suggest that IUs were treated with some respect by providers of health care services. It has been suggested that good program incentives given to providers could influence their behavior towards patients [10]*.*

In our study, IUs of public facilities reported being treated with less dignity than those of private facilities. This observation agrees with earlier reports which found that private providers show more respect for patients’ dignity than public providers [22,25,26]. A cross-country comparative analysis at aggregate level showed countries where education is higher experience higher levels of dignity [22]; however, in a country with low levels of education, we also observed at the individual level that highly educated IUs received more respect by providers than poorly educated IUs. It appears relevant that the NHIS promotes respect for patients’ dignity. One approach to improve client-provider relationships and to encourage improved interactions might be to provide the necessary informational materials to both the IUs and health care providers.

### Communication

As in other parts of the world, communication was found to be of high importance in Nigeria [9,25]. Communication should assure clear patient-provider interactions and Valentine et al. [10] explained that clarity of communication implies that the provider listens carefully to patient’s concerns and explains about an illness’ symptoms, treatment and implications. Furthermore, studies have shown that this communication has to be done in a comprehensive manner which permits the patient to ask follow-up questions [10,31]. However, we found the IUs in our study were not pleased with this domain. Overall, our findings revealed that IUs in the NHIS felt that providers did not listen to them with sufficient concern related to their illness and also did not always give the chance to ask follow-up questions.

We observed that IUs using public facilities reported relatively better communication than those using private facilities. This finding is consistent with earlier observations and implies that private providers are less likely to provide a good chance for communication with patients [9,10,25]. We also found that IUs with lower income were more pleased with the information given by and interactions with providers. A previous study in Nigeria showed that IUs’ knowledge of the NHIS is an important positive determinant of contentment with health care services [17]. In addition, a further possible explanation might be that IUs with high income levels have higher expectations from their health care services.

This study indicates that IUs with a shorter duration of enrolment were more likely to be pleased with communication than those with a longer enrolment in the NHIS. Previous studies have cautioned that poor attitudes of providers and high expectations by IUs could have future adverse consequences on health care services [29]. Our observation that IUs who had been referred to secondary or tertiary care were relatively less pleased with communication by the providers, suggests that improving the referral system might mitigate some of the challenges faced by both IUs and providers in the NHIS.

### Autonomy

IUs identified “autonomy” as weakly important for responsiveness, and this is similar to another related study in Nigeria [25]. Moreover, we found that IUs were least contented with the “autonomy” domain related to NHIS services. Several studies have explained that this “autonomy domain” incorporates the concept of empowerment, where users have the right to medical information, make informed choices and may refuse medical treatment [10,32]. This implies that the providers should involve the patients (and their families where appropriate) in the decision-making process of the treatments [10]. Based on our findings, the IUs were not involved as much as they’d like to be in making decisions regarding their health care treatments. Due to the asymmetry of information between patients and clinicians, the IUs lacked the necessary tools to empower themselves in the decision-making process.

We found that public providers involved IUs less in decision-making than private providers. However, males were relatively more pleased with the “autonomy” than females. By contrast, IUs with longer duration of enrolment in the NHIS were apparently less pleased with their involvement in the decision-making process of their treatment. This study suggests that the health insurance schemes should encourage patients’ empowerment in health care services. Our findings raise the possibility that IUs who were referred for secondary or tertiary care were less likely to experience autonomy. This is similar to previous studies which found that patients who are referred feel discontent with the “autonomy” rendered by the providers during referrals [9,10].

### Choice of provider

We found the “choice of provider” was the third most valued of the responsiveness domains. Valentine et al. [10] explains that this “choice of provider” domain assures that users have the choice of consulting the same providers if desired, while consulting different providers in the event of dissatisfaction. In essence, providers who know that patients have a choice of provider tend to offer quality health care services to their patients with empathy [10]. Although services in the Nigerian NHIS are purchased through a mix of public and private providers, and IUs theoretically have the option to choose their health care providers, this has not been effective in practice [12,14]. Our findings confirm that, despite their displeasure, IUs encountered difficulties in choosing their providers. Further studies have shown delays experienced by providers in receiving authorization to offer services to clients, as well as to receive approval to refer patients across the levels of primary, secondary and tertiary care [24].

The type of facility was found to influence the “choice of provider”. This agrees with previous studies in that users of private providers are usually more contented with their choice of providers than those with public providers [9,25,26]. This evidence from our study suggests that the NHIS might encourage competition among public and private health care providers and concurrently promote IUs’ choice of providers in the event of dissatisfaction. Our findings revealed that low income IUs were better pleased with their “choice of provider” than those with high income status. By contrast, those practicing polygamy were more discontent with the “choice of provider” domain. A possible explanation might be that some expectations of polygamous IUs, such as the inclusion of all their family members in the NHIS, are not fulfilled [33].

Short duration of enrolment was found to increase the likelihood of a highly contented “choice of provider” response compared to a longer duration of enrolment. Possibly, the longer people were enrolled in the NHIS, the less leverage they had in “choice of provider”. We also found that IUs who were referred to secondary or tertiary care level tended to be displeased with the “choice of provider”, suggesting that the NHIS and health care providers might reasonably facilitate IUs’ choice of providers, but concurrently also avoid over-congestion by IUs of a particular facility or provider.

### Quality of basic facilities

Our study agrees with others in finding that “quality of basic facilities” is important to patients in their experience of responsiveness from health care services [9,10,22,25,26]. Our findings indicate that users of the NHIS were most pleased with this domain. According to the World Health Report (2000) [9] and Valentine et al. [10], quality of basic facilities implies that health facilities have clean waiting rooms, toilet facilities, examination rooms and surroundings. We found that there are variations between the IUs characteristics and their contentment with the quality of basic facilities of the providers.

The IUs from public providers were more likely to be displeased with the quality of basic facilities than those from private providers. This implies that there is still the need for public providers to improve the quality of their basic facilities so that they can retain and attract clients. Previous studies in Nigeria and South-Africa have shown that patients who visit private health facilities are better pleased with the quality of basic facilities as compared to the public health facilities [25,26]. We also found that IUs with lower education were displeased with the “quality of basic facilities” of health services.

This study on responsiveness of health care services within the NHIS was facility-based, but we further employed the household tracking approach (as a mop-up) to trace the IUs. During our data analysis, due to the limitations of ordinal logistic regression that inflates the effect of explanatory variables on the outcome variables, we used the generalized ordered logit regression that fits other models with less restriction and reliable interpretations [27,28,30]. Further similar studies should be conducted in other parts of Nigeria and LMICs to explore variations in responsiveness domains of the NHIS related to health care services. This future research should, therefore, concentrate on the investigation of possible regional variations in and comparisons between responsiveness of health care services of States in Nigeria and regions in LMICs.

## Conclusions

The assessment of the responsiveness of health care services within the NHIS was found to be useful in investigating the scheme’s implementation. The domains of “autonomy”, “communication” and prompt attention” were identified as crucial areas to improve the perceived responsiveness of healthcare services. Reform strategies should center on these weak domains, taking into account the characteristics of users most likely to influence their expectations, experience and perception of esponsiveness. Generally, health care providers’ politeness toward clients, decreased waiting at hospitals using specified appointment times, and increased availability of hospital personnel at all times will help improve health care services in the insurance scheme.

## Competing interests

The authors declare that they have no competing interest.

## Authors’ contributions

SM, HD, AS, JLB and RS conceptualized and designed the study protocols. SM and HD carried out the field study work. SM and JL analysed and interpreted the data, while HD gave further inputs in analysis and interpretation of data. SM drafted the manuscript. HD, AS, JL and RS critically reviewed the findings. HD and RS guided the analytical strategy, the presentation of results, and policy conclusion. All authors read and approved the final manuscript.

## Pre-publication history

The pre-publication history for this paper can be accessed here:

http://www.biomedcentral.com/1472-6963/13/502/prepub

## Supplementary Material

Additional file 1: Table S7 Probability values on the parallel line assumption considering a 0.05 level of significance in generalized ordered logit regression for responsiveness domains (a significant test statistic indicates that the parallel regression assumption has been violated). **Table S8.** Overall statistics from the generalized ordered logit regression for responsiveness domains related to Tables 5 and 6. Click here for file
