# Supplementary material for: Assessing responsiveness of health care services within a health insurance scheme in Nigeria: users’ perspectives
Source: BMC Health Serv Res. 2013 Dec 1;13:502. doi: 10.1186/1472-6963-13-502 (PMC4220628; doi:10.1186/1472-6963-13-502)
Supplement: Additional file 1: Table S7 — Probability values on the parallel line assumption considering a 0.05 level of significance in generalized ordered logit regression for responsiveness domains (a significant test statistic indicates that the parallel regression assumption has been violated). Table S8. Overall statistics from the generalized ordered logit regression for responsiveness domains related to Tables 5 and 6. [file 1472-6963-13-502-S1.docx]

**Supplementary file for statistical testings of generalized ordered logit regression**

**Supplementary Table 7: Probability values on the parallel line assumption considering a 0.05 level of significance in generalized ordered logit regression for responsiveness domains (a significant test statistic indicates that the parallel regression assumption has been violated)**

|  | Prompt attention | | Dignity | | Communication | | Autonomy | | Choice of provider | | Quality of facilities | |
| --- | --- | --- | --- | --- | --- | --- | --- | --- | --- | --- | --- | --- |
| Independent Variables | Constraints for parallel lines imposed | p-value | Constraints for parallel lines imposed | p-value | Constraints for parallel lines imposed | p-value | Constraints for parallel lines imposed | p-value | Constraints for parallel lines imposed | p-value | Constraints for parallel lines imposed | p-value |
| Type of facility visited | Yes | 0.1122 | Yes | 0.9246 | No | 0.00471 | No | 0.00000 | Yes | 0.1775 | Yes | 0.6920 |
| Age | Yes | 0.3046 | Yes | 0.0650 | Yes | 0.0902 | No | 0.00030 | Yes | 0.1380 | Yes | 0.5455 |
| Sex | Yes | 0.4741 | Yes | 0.3725 | Yes | 0.7722 | Yes | 0.13398 | No | 0.00002 | Yes | 0.1686 |
| Educational status | Yes | 0.1619 | Yes | 0.2716 | Yes | 0.1884 | No | 0.01900 | Yes | 0.2847 | Yes | 0.1387 |
| Monthly income level | Yes | 0.2940 | Yes | 0.0748 | Yes | 0.1920 | Yes | 0.15305 | Yes | 0.4262 | Yes | 0.2136 |
| Type of marital status | Yes | 0.6777 | Yes | 0.9462 | Yes | 0.1501 | Yes | 0.18208 | Yes | 0.2328 | Yes | 0.5568 |
| Duration of enrolment | No | 0.02636 | Yes | 0.1281 | No | 0.01318 | Yes | 0.4483 | Yes | 0.1942 | Yes | 0.3137 |
| 12months visits to HCPs | No | 0.04161 | Yes | 0.1103 | No | 0.00005 | Yes | 0.4223 | No | 0.00098 | No | 0.01292 |
| Referral after enrolment | No | 0.03192 | No | 0.02159 | No | 0.03431 | No | 0.03091 | No | 0.00038 | Yes | 0.1976 |
|  |  |  |  |  |  |  |  |  |  |  |  |  |

**Supplementary Table 8: Overall statistics from the generalized ordered logit regression for responsiveness domains related to Table 5 and 6**

| Domains | Prompt attention | Dignity | Communication | Autonomy | Choice of provider | Quality of facilities |
| --- | --- | --- | --- | --- | --- | --- |
| Wald *X^2^* | 88.27 | 54.39 | 124.04 | 99.16 | 115.55 | 112.42 |
| P > *X^2^* | 0.0000 | 0.0000 | 0.0000 | 0.0000 | 0.0000 | 0.0000 |
| R^2^ | 0.0453 | 0.0296 | 0.0647 | 0.0449 | 0.0564 | 0.0651 |
| N | 796 | 796 | 796 | 796 | 796 | 796 |
|  |  |  |  |  |  |  |
| Model test | An insignificant test statistic indicates that the final model does not violate the proportional odds/ parallel lines assumption | | | | | |
| *X^2^* | 21.47 | 28.94 | 21.77 | 23.91 | 26.08 | 25.36 |
| P > *X^2^* | 0.2565 | 0.2226 | 0.1141 | 0.2752 | 0.0980 | 0.3863 |
|  |  |  |  |  |  |  |
